# Plasma Orexin-A Levels in Patients With Schizophrenia: A Systematic Review and Meta-Analysis

**DOI:** 10.3389/fpsyt.2022.879414

**Published:** 2022-05-25

**Authors:** Shaoli Li, Ruili Zhang, Shaohua Hu, Jianbo Lai

**Affiliations:** ^1^Department of Psychiatry, The First Affiliated Hospital, Zhejiang University School of Medicine, Hangzhou, China; ^2^Department of Medical Oncology, The Second Affiliated Hospital, Zhejiang University School of Medicine, Hangzhou, China; ^3^The Key Laboratory of Mental Disorders' Management in Zhejiang Province, Hangzhou, China; ^4^Brain Research Institute of Zhejiang University, Hangzhou, China; ^5^Zhejiang Engineering Center for Mathematical Mental Health, Hangzhou, China; ^6^Department of Neurobiology, NHC and CAMS Key Laboratory of Medical Neurobiology, School of Brain Science and Brian Medicine, and MOE Frontier Science Center for Brain Science and Brain-Machine Integration, Zhejiang University School of Medicine, Hangzhou, China

**Keywords:** schizophrenia, orexin-A, meta-analysis, medication, cognitive deficits

## Abstract

**Background:**

Orexins are polypeptides regulating appetite, sleep-wake cycle, and cognition functions, which are commonly disrupted in patients with schizophrenia. Patients with schizophrenia show a decreased connectivity between the prefrontal cortex and midline-anterior thalamus, and orexin can directly activate the axon terminal of cells within the prefrontal cortex and selectively depolarize neurons in the midline intralaminar nuclei of the thalamus. To address the relationship between orexin and schizophrenia, this study performed a meta-analysis on the alteration of plasma orexin-A levels in patients with schizophrenia.

**Method:**

We searched eligible studies in PubMed, Embase, Cochrane, and China National Knowledge Infrastructure (CNKI) from 1998 to September 3, 2021. A total of 8 case-control studies were included in the meta-analyses, providing data on 597 patients with schizophrenia and 370 healthy controls. The Stata version 16.0 software was used to calculate the Hedges's adjusted g with 95% confidence intervals (CI).

**Results:**

The plasma orexin-A levels were not altered in subjects with schizophrenia (*n* = 597) when compared to healthy controls (*n* = 370). Subgroup analyses of gender (male and female vs. only male), country (China vs. other countries), medication (medication vs. non-medication), and the measurement of plasma orexin-A (Enzyme-linked immunosorbent assay vs. radioimmunoassay) revealed heterogeneity ranging from 30.15 to 98.15%, but none showed a significant alteration of plasma orexin-A levels in patients with schizophrenia. Heterogeneity was lower in the other countries and radioimmunoassay subgroup, while other subgroups remained to be highly heterogeneous. No significant evidence of publication bias was found either in Begg's test or the Egger's test.

**Conclusion:**

The present meta-analysis indicated that patients with schizophrenia did not show abnormal plasma levels of orexin-A.

**Systematic Review Registration:**

https://www.crd.york.ac.uk/prospero/display_record.php?ID=CRD42021283455, identifier: CRD42021283455.

## Introduction

Schizophrenia is a severe and chronic mental disorder with a heterogenous combination of symptoms. The lifetime prevalence of schizophrenia is about 1% worldwide (1). A meta-analysis revealed that patients with schizophrenia have a 5 to 10% lifetime risk of death by suicide and an average decrease of 15-year life expectancy than the general population (2). The etiology of schizophrenia is complicated and predominantly linked to the interactions between the genetic susceptibility and environmental factors, such as early life stressors (3). And its pathophysiology remains to be elusive. One of the main hypotheses of schizophrenia refers to the hyperactivity in subcortical dopaminergic functions, and accumulative evidence suggests that alterations in serotonergic, glutamatergic, and gamma-aminobutyric acid are closely related to the onset and development of schizophrenia (4). Clinical pharmacotherapy of schizophrenia is mostly based on the dopaminergic hypothesis, but most antipsychotics show limited efficacy in improving negative and cognitive symptoms of the disease (5). Therefore, the development of novel treatment strategies calls for better understanding of the pathophysiology of schizophrenia (4).

Orexins (also named hypocretin) are neuropeptides synthesized by orexin-containing neurons in the lateral hypothalamus and perifornical regions (6). These neurons send projections to many brain areas, including the prefrontal cortex, locus coeruleus, hippocampus, hypothalamus, and dorsal raphe nucleus (7). Orexins commonly are present in two forms, orexin-A (hypocretin-1) and orexin-B (hypocretin-2). They mediate signals by binding to the corresponding orexin-1 and orexin-2 receptors. Orexin-A exhibits similar affinity for these two receptors, while orexin-B preferentially binds to the orexin-2 receptor (8).

Orexins regulates various physiological processes, such as cognition, sleep-wake cycle and energy balance (9). Previous studies proposed that orexins and orexin receptor agonists might play a role in alleviating cognitive deficits in patients with schizophrenia (10). Preclinical data also suggested that drugs activating orexin neurons may be useful in treating the negative symptoms such as cognitive deficits in schizophrenia (11–13). As a major type of orexin in the brain, orexin-A is highly lipophilic, stable and resistant to degradation, and can rapidly cross the blood-brain-barrier via simple diffusion. However, orexin-B has low lipophilicity and is rapidly metabolized in the peripheral circulation (14). Therefore, most studies focused on exploring the plasma orexin-A levels in specific diseases (15–17). The levels of orexin-A may be influenced by various factors. For example, previous study showed that orexin-A levels in the hypothalamus of female rats were elevated compared to male rats (18). Nicholas et.al found a decrease in orexin expression with normal human maturation and aging (19). In addition, several animal studies indicated that orexin-A was increased in rats exposed to smoke (20, 21). Retrospective studies also found orexin peptides were enhanced by nicotine treatment, which investigated a close correlation between orexin and smoking (22). The exact role of orexins in schizophrenia is still unclear, but there are complex neuroanatomical interactions between orexin-A and the dopamine system in various areas of the brain. For example, orexin-containing projections affect the activity of dopamine neurons and the release of dopamine in the ventral tegmental area (23) and the nucleus accumbens (24), suggesting the potential association between the orexin system and schizophrenia. Several researches have preliminarily explored their relationship by examining the plasma orexin-A level in patients with schizophrenia. Some studies showed that in patients with schizophrenia, an elevated plasma orexin-A level was related to the negative and disorganized symptoms (9, 25). It was also found that the plasma orexin-A levels were associated with risk of metabolic disturbance in patients with schizophrenia (26, 27). However, other studies found that there was no difference in plasma orexin-A levels between patients with schizophrenia and healthy controls (28). These findings appear contradictory and a qualitative synthesis of studies addressing these dissimilarities is needed. To date, no meta-analysis on this topic has been conducted and we aimed to perform a systematic review and meta-analysis to investigate the association between plasma orexin-A levels and schizophrenia.

## Methods and Material

This present study was conducted under the guidance of Preferred Reporting Items for Systematic Reviews and Meta-analyses guidelines (29). This systematic review and meta-analysis has been registered in the PROSPERO (ID: CRD42021283455). The literature search, decisions on inclusion, data extraction and quality control were all performed independently by two of the authors (SL and RZ).

### Search Strategy

We conducted a systematic search for all potentially eligible-English and non-English peer-reviewed articles to avoid language publication bias (30, 31) using English database (PubMed, Embase, the Cochrane Library) and the Chinese database, China National Knowledge Infrastructure (CNKI). No year or country restrictions were used. An independent online search covered the publication period from 1998 (the year when the orexins were discovered) to 3 September, 2021. We applied a combination of Medical Subject Headings and free-text terms, including their variants, to search in PubMed and Cochrane library. The following combination of keywords were used to search: ((((((((Schizophrenia) OR (Schizophrenic Disorders)) OR (Disorder, Schizophrenic)) OR (Disorders, Schizophrenic)) OR (Schizophrenic Disorder)) OR (Dementia Praecox)) OR (“Schizophrenia”[Mesh])) AND ((((((((((((Hypocretins) OR (Hypocretin)) OR (Orexin)) OR (Orexin-B)) OR (Orexin B)) OR (Hypocretin-2)) OR (Hypocretin 2)) OR (Orexin-A)) OR (Orexin A)) OR (Hypocretin-1)) OR (Hypocretin 1)) OR (“Orexins”[Mesh]))). We search in Embase and CNKI using the search terms *schizophrenia* and *orexin*. Additionally, reference lists of potentially eligible publications were reviewed.

### Inclusion Criteria and Exclusion Criteria

The inclusion criteria were as follows: (1) adult subjects with schizophrenia, as defined by the Diagnostic and Statistical Manual of Mental Disorders, the 4^th^ edition (*DSM-IV*) or International Classification of Diseases-10 (ICD-10); (2) the controls were healthy individuals; (3) studies assessing circulating serum or plasma orexin-A in human blood samples. Exclusion criteria were as follows: (1) non-original articles (reviews, meta-analyses, commentaries, letters, conference abstracts and editorials); (2) studies without healthy controls; (3) studies in animal models; (4) samples independent of plasma or serum; (5) patients comorbid with physical diseases (hypertension, obesity, Huntington's disease, amyotrophic lateral sclerosis, and multiple sclerosis, neurodegenerative diseases such as Alzheimer's disease) that may affect orexin levels and other mental illness. The decision on whether to include studies in the meta-analysis was made according to the above criteria, and a consensus was reached among the authors on those decisions.

### Data Extraction and Quality Evaluation

Mean and standard deviation or the percentage of the events were extracted for the following variables: age, gender, body mass index (BMI), smoking status, medication, orexin-A levels, and the Positive and Negative Syndrome Scale (PANSS) scores. We also extracted the first author's name, publish year, the country where the study was performed, and the study design. Newcastle-Ottawa Scale (NOS) was used to assess the quality of studies. NOS is an eight-item scale that are categorized into 3 groups: the selection of the study groups, the comparability of the groups and the ascertainment of either the exposure or outcome of interest for case-control or cohort studies, respectively. The total score of NOS is 9 (32). Studies with higher quality got higher scores. If there was any disagreement, discussion and decisions were made by all authors.

### Statistical Analysis

All statistical analyses were performed with Stata version 16.0 software (Stata Corp, College Station, TX, USA). Due to the inconsistent measurement methods of the eight studies, standardized mean difference estimated of the difference in orexin-A levels between subjects with schizophrenia and healthy controls was used as the effect size (ES), which was adjusted by Hedges's g to calculate an unbiased ES for small sample sizes (33, 34). The 95% confidence interval (CI) of the ES was also computed with the random-effect model to assess the pooled difference on plasma orexin-A levels between patients with schizophrenia and the controls. The I^2^ was selected to assess the heterogeneity between studies (I^2^ values of 0, 25, 50, and 75% represent no, low, moderate, and high heterogeneity, respectively) (34). When the heterogeneity was >50%, a random-effect model was selected, or otherwise, a fixed-effect model was used (35). Subgroup analyses were performed by gender, country, medication and the measurement of plasma orexin-A levels. Sensitivity analysis was performed by omitting one study at a time to detect any significant change in the results. We used Begg's rank correlation test and Egger's regression asymmetry test to evaluate publication bias. All reported probabilities (*P*-values) were two-sided, and *P* < 0.05 was considered statistically significant.

## Results

### Characteristics of Studies

**Figure 1:**
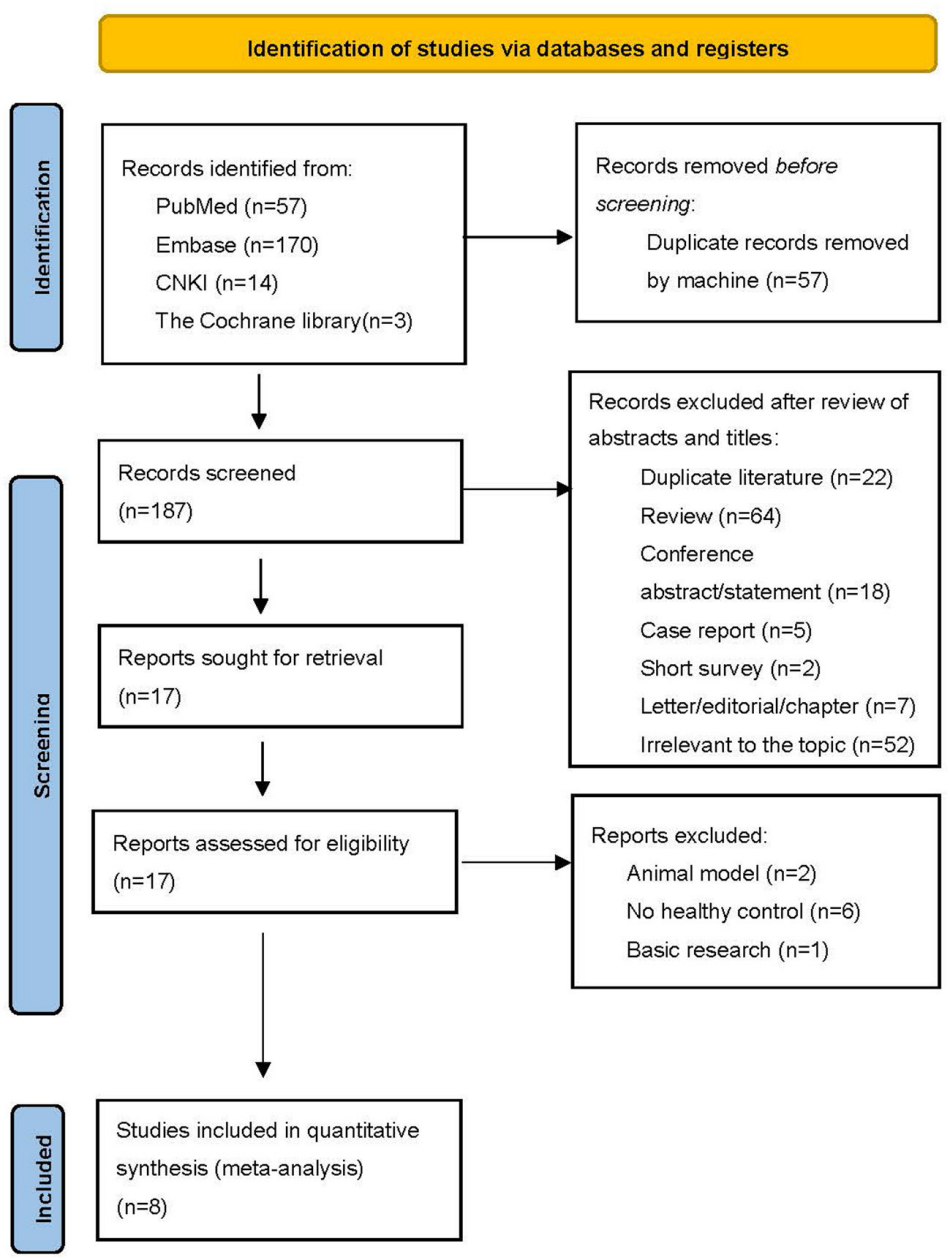
Flow diagram of literature search.

A total of 244 records were identified, of which 57 articles were screened as duplicates by the machine. Then two authors (SL, and RZ) independently read the abstracts and titles of the remaining 187 articles and excluded 170 articles (including duplicate literature, conference abstract, review, case report, letter and other reasons). Eight studies were finally included in the systematic review and meta-analysis (Figure 1). General detailed characteristics (age, gender, BMI, smoking status, and medication) of eligible studies were shown in Table 1. These studies determined the plasma orexin-A levels in 597 patients with schizophrenia and 370 healthy controls. The participants in most studies were Chinese except one study whose participants were from Japan and one study from Turkey, which may influence the final results. In most studies, patients with schizophrenia were matched for age and gender, except for one study (28), in which patients were significantly younger than healthy controls. Two studies only included male subjects. In addition, five studies focused on patients with first-episode schizophrenia (*n* = 231), while patients with schizophrenia in the remaining three studies had received relevant medications (*n* = 366). Enzyme-linked immunosorbent assay (ELISA) was used to measure the plasma orexin-A level in five articles, and radioimmunoassay (RIA) was used in the remaining three studies.

**Table 1:** Characteristics of included studies in the meta-analysis for plasma orexin-A levels.

			**Schizophrenia group**				**Control group**						
**References**	**Study design**	**Country**	**N(M/F)**	**Age,years** **(mean±SD)**	**BMI (kg/m^**2**^)**	**Smoking status**	**N(M/F)**	**Age,years** **(mean±SD)**	**BMI (kg/m^**2**^)**	**Smoking status (%)**	**Orexin measure**	**Medication**	**PANSS** **score**
Tsuchimine et al. (28)	Case-control	Japan	80(38/42)	36(45)0.8 ± 11.2	23.2 ± 4.4	29.5	80(32/48)	47.0 ± 14.2	21.9 ± 3.0	12.7	ELISA	Typical antipsychotic medication (65.0%) Atypical antipsychotic medication (17.5%) Antidepressant Medication (15.0%) Benzodiazepine medication (21.3%) Lithium medication (2.5%) Benzodiazepine medication (1.3%) Varproric acid medication (5.0%) Lamotrigine medication (2.5%)	Positive symptom (13.8 ± 5.3) Negative symptom (15.5 ± 6.2) General (31.3 ± 8.1)
Sun et al. (26)	Case-control	China	13(13/0)	22.54 ± 3.84	/	/	15(15/0)	22.20 ± 2.14	/	/	RIA	First episode, no medication	Total: 108.23 ± 11.03
Lu et al. (36)	Case-control	China	61(27/34)	26.4 ± 10.9	/	/	82(38/44)	27.3 ± 9.4	/	/	ELISA	First episode, no medication	/
Chien et al. (9)	Case-control	China	127(53/74)	38.8 ± 10.5	/	15.7	34(20/14)	37.1 ± 10.6	/	18.6%	RIA	Antipsychotic medication	Total: 52.34 ± 13.86
Chen et al. (27)	Case-control	China	159(77/82)	41.13 ± 9.28	25.08 ± 4.50	20.8	60(29/31)	41.10 ± 9.65	24.00 ± 6.11	11.7%	ELISA	Clozapine medication(109) Less obesogenic Aps (50)	/
Basoglu et al. (37)	Case-control	Turkey	20(20/0)	21.2 ± 0.75	22.0 ± 2.2	65.0	22(22/0)	21.7 ± 1.1	22.4 ± 2.0	77.3%	ELISA	First episode, no medication	Total: 95.2 ± 14.8
Zhang et al. (38)	Case-control	China	61(22/39)	34.22 ± 11.86	22.82 ± 3.5	/	37(14/23)	35.37 ± 10.45	22.85 ± 3.64		RIA	First episode, no medication	/
Li et al. (39)	Case-control	China	76(27/49)	34.59 ± 3.68	22.46 ± 2.37		40(15/25)	35.11 ± 3.53	22.59 ± 2.48		ELISA	First episode, no medication	/

*M, male; F, female; BMI, Body mass index; PANSS, Positive and Negative Syndrome Scale; ELISA, Enzyme-linked immunosorbent assay; RIA, Radioimmunoassay*.

### Quality Evaluation

According to the NOS, all the included studies had clear diagnostic criteria for schizophrenia. All the healthy controls had no history of mental disorders. There was no difference in gender between the healthy group and the schizophrenia group. Consequently, the two groups were comparable. The majority of included studies did not mention the non-response rate, and only one study Basoglu et al. mentioned a low non-response rate for the two groups (37). The total quality of eight studies was at least 6, suggesting that the quality of included studies was high (shown in Table 2).

**Table 2:** Results of quality assessment using the Newcastle–Ottawa Scale for case-control studies.

	**Selection**					**Exposure**			
**References**	**Adequate definition of cases**	**Represen-tativeness of the cases**	**Selection of con- trols**	**Definition of con-** **trols**	**Comparability Control for** **important factor ^a^**	**Ascertain-** **ment of** **exposure**	**Same method of ascertain- ment for cases and controls**	**Non-** **response** **rate**	**Scores**
Tsuchimine et al. (28)	**⋆**	**⋆**	–	**⋆**	**⋆**	**⋆**	**⋆**	–	6
Sun et al. (26)	**⋆**	**⋆**	**⋆**	**⋆**	**⋆⋆**	**⋆**	**⋆**	–	8
Lu et al. (36)	**⋆**	**⋆**	**⋆**	**⋆**	**⋆⋆**	**⋆**	**⋆**	–	8
Chien et al. (9)	**⋆**	**⋆**	**⋆**	**⋆**	**⋆⋆**	**⋆**	**⋆**	–	8
Chen et al. (27)	**⋆**	**⋆**	–	**⋆**	**⋆**	**⋆**	**⋆**	–	6
Basoglu et al. (37)	**⋆**	–	–	**⋆**	**⋆**	**⋆**	**⋆**	**⋆**	6
Zhang et al. (38)	**⋆**	**⋆**	**⋆**	**⋆**	**⋆⋆**	**⋆**	**⋆**	–	8
Li et al. (39)	**⋆**	**⋆**	**⋆**	**⋆**	**⋆**	**⋆**	**⋆**	–	7

a *A maximum of two stars can be allotted in this category, one for age, the other for other controlled factors*.

### Meta Analyses

**Figure 2:**
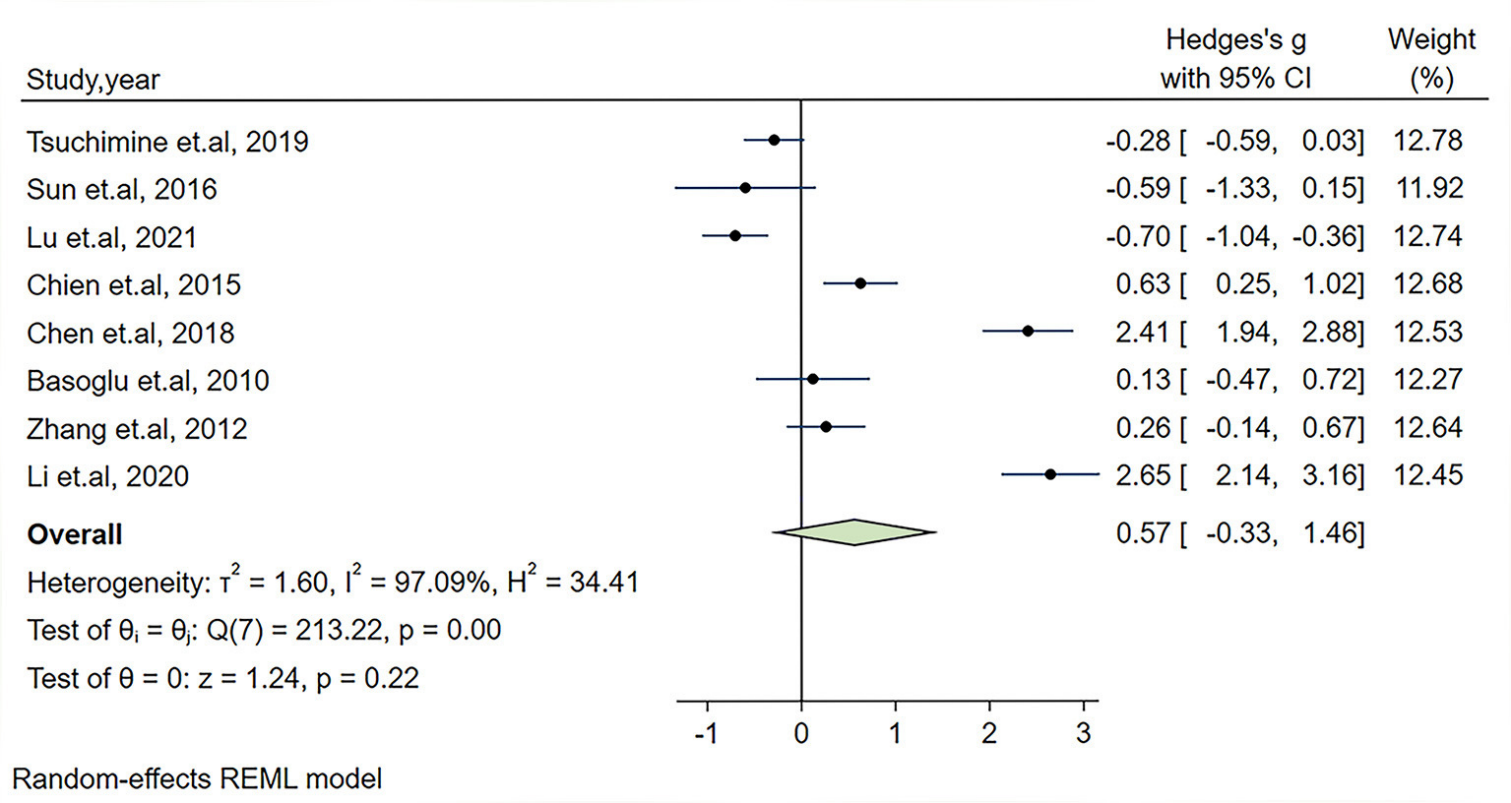
Forest plot of Hedges's g with corresponding 95% confidence interval (CI) of studies on the association between plasma orexin-A levels and schizophrenia.

In our meta-analysis, the Hedges's g and its corresponding 95% CI was calculated based on the extracted 8 studies compassing 597 patients with schizophrenia and 370 healthy controls. Because an obvious heterogeneity across studies existed (I^2^ = 97.07%, *P* < 0.01), a random-effect model was used in our analysis. The combined results of the overall comparison revealed that there was no significant change in plasma orexin-A level in the schizophrenia group compared with healthy controls (Hedges's g = 0.57, 95% CI: −0.33,1.46, *P* = 0.22, see Figure 2).

### Subgroup Analyses

**Figure 3:**
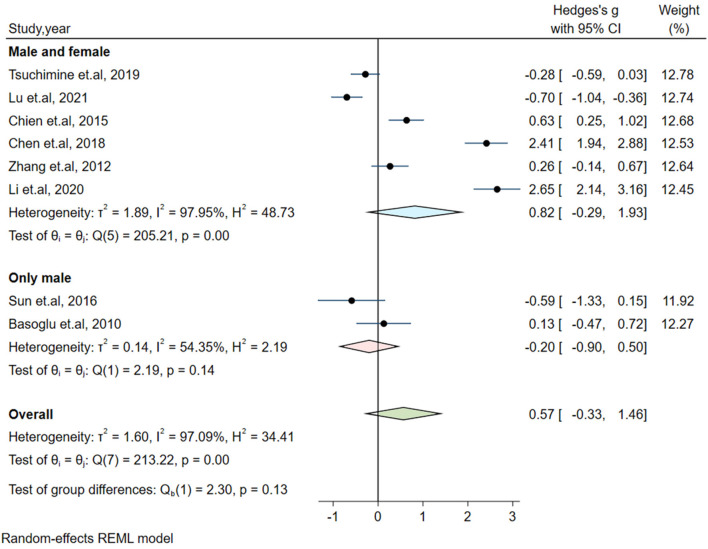
Subgroup analysis of plasma orexin-A levels in patients with schizophrenia compared to healthy controls (based on gender).

**Figure 4:**
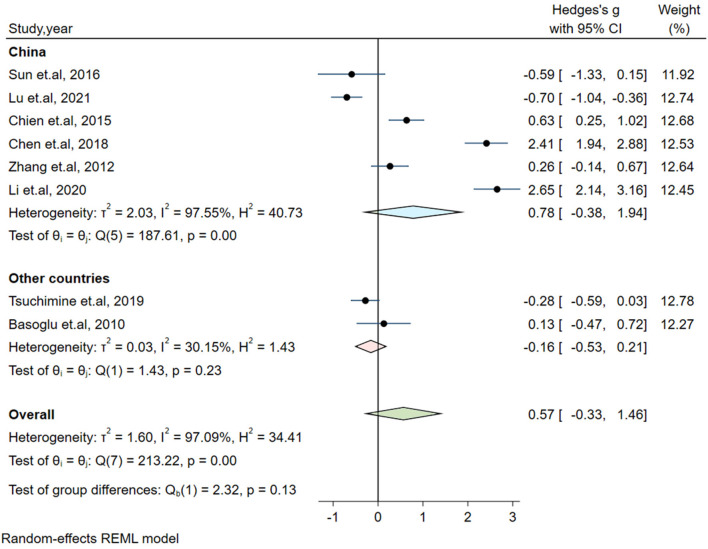
Subgroup analysis of plasma orexin-A levels in patients with schizophrenia compared to healthy controls (based on country).

**Figure 5:**
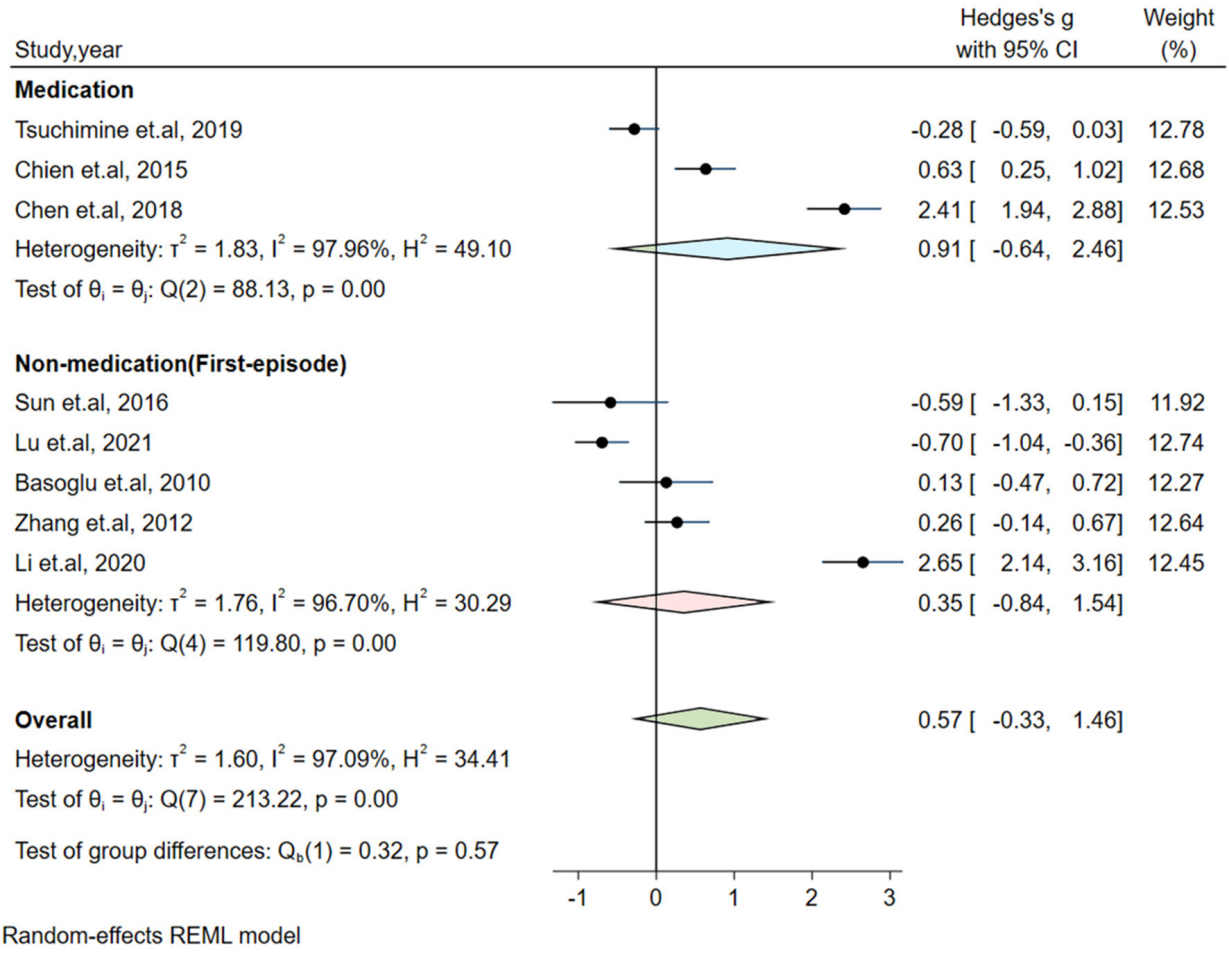
Subgroup analysis of plasma orexin-A levels in patients with schizophrenia compared to healthy controls (based on medication).

**Figure 6:**
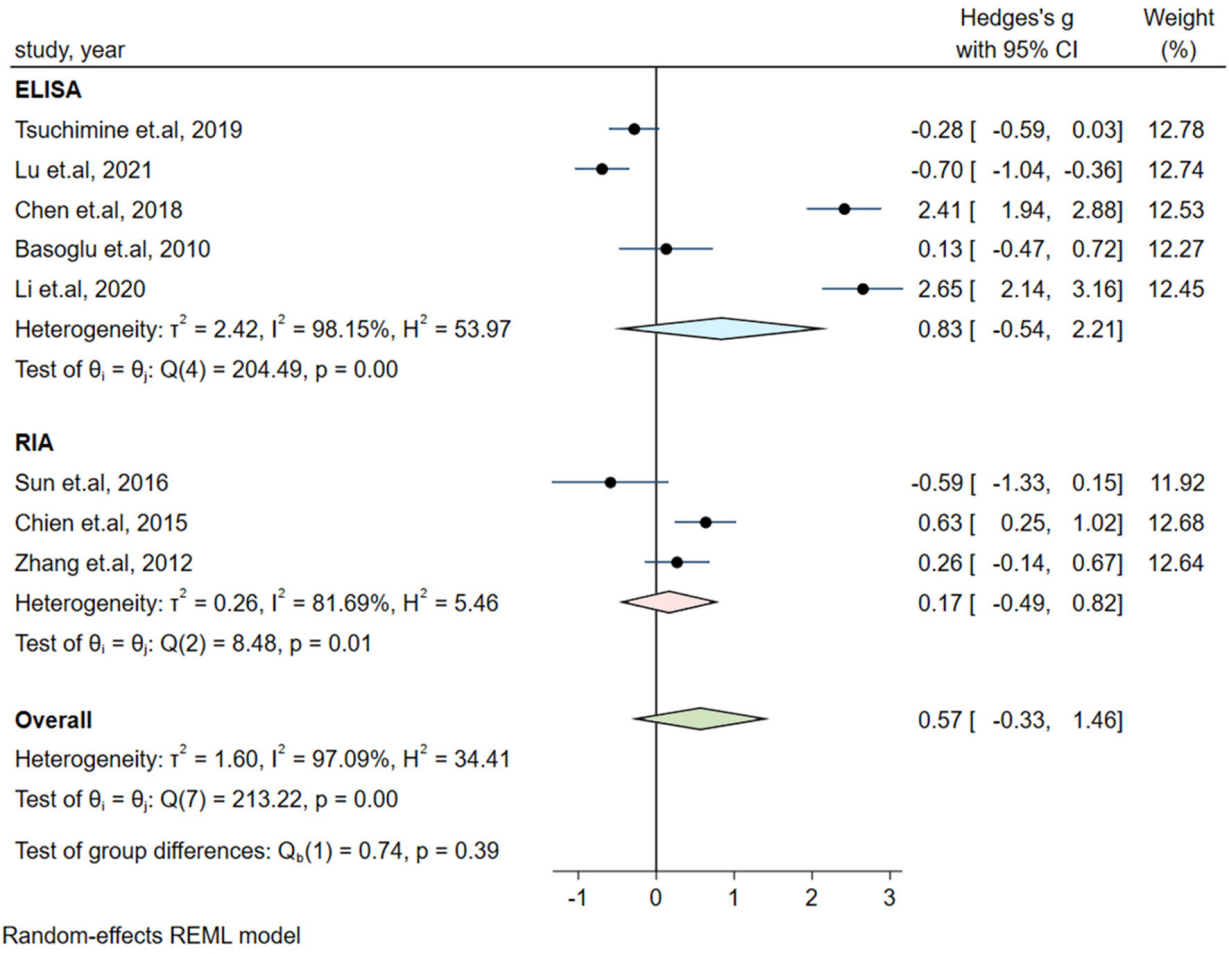
Subgroup analysis of plasma orexin-A levels in patients with schizophrenia compared to healthy controls (based on the measurement of plasma orexin-A).

Significant heterogeneity could influence the results. Therefore, a series of approaches were applied to reduce the impact of heterogeneity. Subgroup analyses were carried out based on gender (male and female, only male), country, medication and the measurement of plasma orexin-A levels (ELISA and RIA), and results of subgroup analysis based on gender showed moderate heterogeneity (I^2^ = 54.35%, *P* = 0.14) in the male group, but the plasma orexin-A was not altered in the two subgroups (see Figure 3). Heterogeneity decreased in the other countries subgroup (I^2^ = 30.15%, *P* = 0.23) and the RIA subgroup (I^2^ = 81.69%, *P* = 0.01), while other subgroups remained to be highly heterogeneous (see Figures 4–6).

### Influence Analysis and Publication Bias

**Figure 7:**
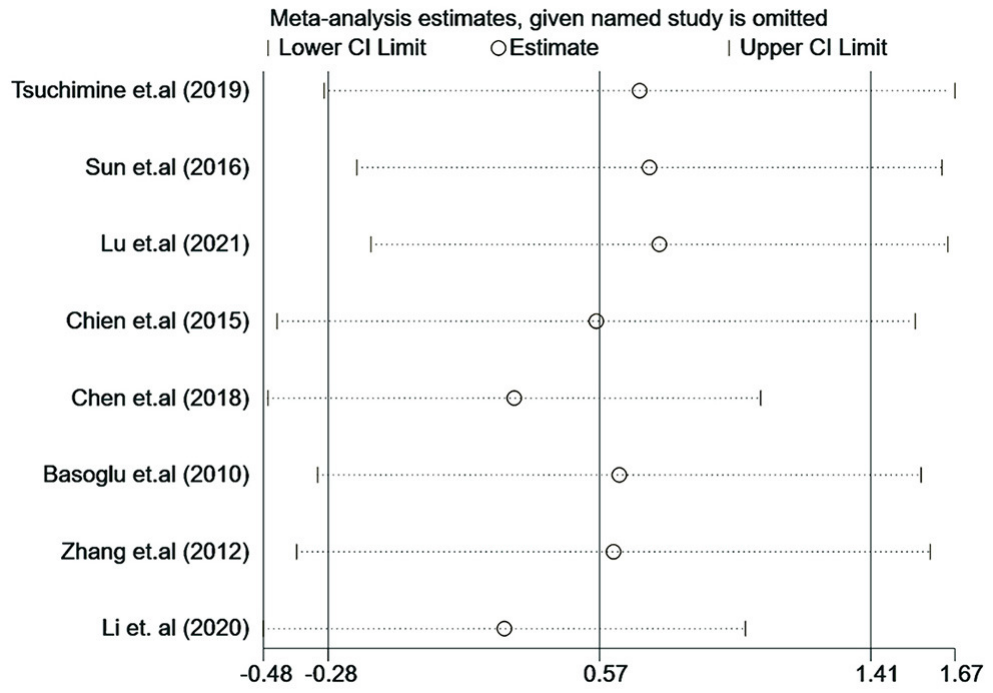
Influence analysis of individual study on the pooled estimate for studies on the association between plasma orexin-A levels and schizophrenia.

Influence analysis showed that no study had an excessive influence on the plasma orexin-A level between the patients with schizophrenia and healthy controls (see Figure 7). Moreover, there was no evidence of significant publication bias examined by the Eegg's test (*P* = 0.917) or the Bgger's test (*P* = 0.536).

## Discussion

To our knowledge, this is the first meta-analysis to investigate the relationship of plasma orexin-A levels between patients with schizophrenia and healthy controls. The included studies were mainly from the East Asian region, and more than half of the articles explored plasma orexin-A levels in patients with first-episode schizophrenia. The results suggested that the plasma orexin-A levels were not altered in patients with schizophrenia compared to healthy individuals.

In our included studies, most patients with schizophrenia had cognitive deficits, including impairment in learning, memory, and executive functions, which were positively correlated with the plasma orexin-A levels (9, 39). Patients with schizophrenia had a dystrophic change in the dendrites of prefrontal cortex pyramidal cells, and their functional connectivity between the prefrontal cortex and midline-anterior thalamus was decreased, which contributed to the cognitive deficits (40–44). Notably, animals' studies suggested that orexin may act on the ascending arousal system, which consists of thalamocortical projection to the prefrontal cortex and regulates our attention and awareness. By sending projection to associated brain regions, orexin neurons can selectively depolarize neurons in the midline-intralaminar nuclei of the thalamus, and directly activate the axon terminal of these cells within the prefrontal cortex (40, 45). Altogether, these findings suggested that orexin may be involved in cognitive deficits in patients with schizophrenia.

In addition, an animal study revealed that cognitive deficits caused by orexin deficiency were gender-dependent (46). Previous evidence also showed hyperactivity of the orexin system in females (47). But our meta-analysis did not find any significant difference in plasma orexin-A either in males or females. Therefore, further studies are needed to explore the interrelationship among the plasma orexin-A levels, gender and cognitive deficits in patients with schizophrenia.

The included studies showed that most patients with schizophrenia had cognitive deficits, including impairment in learning, memory, and executive functions. Multiple studies have shown that patients' orexin-A levels were positively correlated with the negative symptoms, especially cognitive function (9, 39). In general, common antipsychotics mainly target the patient's positive symptoms, but most have a relatively mild effect on negative and cognitive symptoms (48). In our meta-analysis, most studies focused on the patients with first-episode schizophrenia, and the remaining studies included patients who had a previous history of medication. These patients were primarily treated with typical antipsychotic drugs. Notably, our subgroup analysis showed no significant difference in orexin-A levels either in the first-episode or medication group, which may help to explain why common antipsychotics have a poor effect on patients' cognitive functions.

In our study, of the eight included studies, four studies showed a higher plasma orexin-A in patients with schizophrenia compared with controls, while three articles found no significant difference in the plasma orexin-A between the two groups. The remaining study even showed that the plasma orexin-A levels were lower in patients with schizophrenia. Meta-analysis showed no significant difference in plasma orexin-A levels between patients with schizophrenia and healthy controls, which may be partially explained by the inconsistent methodology across the different studies. For example, previous studies have indicated that BMI may be associated with the levels of orexin-A (25, 49). However, a lack of data on the participants' BMI in three studies may influence the results. Besides, although the remaining five studies showed no difference in BMI between patients with schizophrenia and healthy controls, difference still existed in the average value of BMI across the studies, which may also contribute to the inconsistent results.

An animal study revealed that cognitive deficits caused by orexin deficiency were gender-dependent (46). Previous evidence also showed hyperactivity of the orexin system in females (47). But our meta-analysis did not find any significant difference in plasma orexin-A either in males or females. Therefore, further studies are needed to explore the interrelationship among the plasma orexin-A levels, gender and cognitive deficits in patients with schizophrenia. Moreover, common antipsychotics mainly target at the patient's positive symptoms, but show a relatively mild effect on negative and cognitive symptoms (48). In our meta-analysis, most studies focused on the patients with first-episode schizophrenia, and the remaining studies included patients who had a previous history of medication. These patients were primarily treated with typical antipsychotics and our subgroup analysis showed no significant difference in orexin-A levels either in the first-episode or medication group, which may help to explain why common antipsychotics have a poor effect on patients' cognitive functions.

Some inherent limitations should be mentioned in this meta-analysis. First, only eight studies were included in the final analysis. And most of these studies were from the East Asian region, which may have an influence on the final results to some extent. Therefore, more studies were warranted to draw a more robust conclusion. Second, the sample size of several studies was relatively small and less than 100. Third, high heterogeneity across studies was detected in the present study, and the results of subgroup analyses failed to explain the source of heterogeneity. In addition, due to the lack of detailed information such as BMI, smoking status and PANSS scores in several studies, subgroup analyses were conducted based on available data. Last but not least, in our study, orexin-A levels were measured by RIA or ELISA. While RIA is considered to be the standard for quantifying orexin in cerebrospinal fluid, the accuracy of RIA or ELISA remains a controversial topic (50–52). More importantly, previous studies suggested that RIA and ELISA might recognize orexin-A breakdown products, even hydrolyzed peptides that have little in common with active orexin receptor ligands (53, 54). Thus, it is of great significance to explore more advanced methods for determining plasma orexin-A levels.

## Conclusion

In conclusion, our meta-analysis did not show any significant alteration in plasma orexin-A levels between patients with schizophrenia and healthy controls. However, given that various factors may influence the orexin levels, better designed clinical studies with larger sample size and preclinical studies are needed to draw a clear conclusion on the relationship between orexin and schizophrenia.

## Data Availability Statement

The original contributions presented in the study are included in the article/supplementary material, further inquiries can be directed to the corresponding author/s.

## Author Contributions

SH and JL conceived and designed the study. SL conducted the literature search and wrote the first draft of the manuscript. SL and RZ conducted the data extraction and quality assessment and conducted statistical analyses under the supervision of JL. All authors contributed to the article and approved the submitted version.

## Funding

This study was supported by grants from the Program from the Health and Family Planning Commission of Zhejiang Province (Grant Number: 2020KY548) and the Leading Talent of Scientific and Technological Innovation - Ten Thousand Talents Program of Zhejiang Province (Grant Number: 2021R52016).

## Conflict of Interest

The authors declare that the research was conducted in the absence of any commercial or financial relationships that could be construed as a potential conflict of interest.

## Publisher's Note

All claims expressed in this article are solely those of the authors and do not necessarily represent those of their affiliated organizations, or those of the publisher, the editors and the reviewers. Any product that may be evaluated in this article, or claim that may be made by its manufacturer, is not guaranteed or endorsed by the publisher.
